# The Medical Session

**Published:** 1904-09-03

**Authors:** 


### The Hospital.

A Journal of

The Medical Sciences and Hospital Administration.

Vol. XXXVI.—No. 936. SEPTEMBER 3, 1904.

### The Medical Session.

The period of the year has once more arrived at which young men intended for the medical profession, and their parents and guardians for them, are called upon to exercise discretion and to take thought with reference to the place at which the necessary studies are to be conducted. The choice is one which may be of far greater importance than it seems; because, although every school of medicine in the kingdom affords to its alumni the means of completing a student's career, and of satisfying all the requirements of a qualifying examination, it is none the less plain that they are calculated to exert very different degrees and kinds of influence upon his future. A young man who is ambitious, who is well educated, who is conscious of ability, and who possesses the means to wait, will almost certainly look forward to the attainment of that supreme goal of professional aspiration, the post of physician or surgeon to a great metropolitan hospital; and, as nearly all hospitals are naturally inclined to give to their own students a preference over strangers, he will be materially influenced in his choice by any personal or family associations which may lead him to desire attachment to one institution rather than to another. A prospective student who has family connections and local influence in or near any great provincial city may reasonably desire to strengthen and confirm these by studying at its school and hospitals, and, if he acquit himself in such a manner as to sustain or augment their reputation, he will look to his eventual reward in their service and in the confidence of those who have seen the successive steps of his upward course. Where no special claims or ties exist, as in the case of a student who looks forward to succession to an established family practice, or who intends to enter the army or the navy, or who merely seeks to obtain a modest livelihood as a practitioner, the choice of a school is likely to be much influenced by considerations of convenience, of the proximity of friends or relatives, and of expense—all entitled to varying weight in different circumstances, and none of them to be omitted from consideration by ordinary prudence. With reference to these, of course, no general rules can be laid down.

It must not be forgotten that the question of preliminary education, which at present may be said to have been cast into a crucible out of which its final form has yet to come, is likely to exert a constantly increasing influence over the status of the medical practitioners of the future. The determination of the General Medical Council that, so far as their influence may extend, this education shall be improved, is likely, before long, to make a definite impression upon public opinion; and the apparent unwillingness of the great corporations to give effect to the Council's views is not unlikely unduly to depress the value of the licenses or diplomas which these corporations issue, and to exalt—perhaps also somewhat unduly—the value of university degrees. In these circumstances, it would seem the best policy for every student to aim at connecting himself with an university, and so to arrange his career as to obtain or retain the power of securing the titles which it is able to confer.

Whatever may be done in this direction, the remaining and really essential part of the student's course will be almost equally divided between the acquirement of knowledge introductory to medicine and surgery, and the acquirement of knowledge directly applicable to the purposes of the practitioner. The former division, including anatomy, physiology, organic chemistry, and kindred subjects, requires only teachers, books, dissecting rooms, and laboratories, and its pursuit can be undertaken wherever these are furnished, and with a success proportionate to their efficiency. The work of the second division can only be undertaken in the presence of patients, and its success, other things being equal, is likely to bear some proportion to the number of the patients, to the character and variety of the diseases under which they suffer, and to the completeness and many-sidedness alike of the teaching by which they are explained, and of the skill with which they are treated. The larger the field of hospital work, and the more active a generous rivalry between its cultivators, the more certain it is that those who look and learn will enjoy complete participation in the fulness of the harvest.

We have had to point out on former occasions that the weak side of London medical education is a result of the policy under which it has seemed good to the authorities concerned to have a complete medical school attached to every hospital, although the students at some of them have seldom been sufficiently numerous to meet the cost of the necessary equipment. Many of the provincial schools have avoided this error, and, by a coalition between two or more hospitals for the purposes of preliminary scientific teaching, have conducted this in a manner leaving little to be desired. On the other hand, the strength of London has lain in the limitless quantity and the infinite variety of the available clinical material, and in the number of physicians and surgeons constantly utilising its lessons to the utmost for the advantage of those who are to be taught. In such circumstances, and perhaps in such alone, can young men of average capacity become completely delivered from the provincial spirit, and from the tendency to reciprocal admiration which it is perhaps somewhat too prone to cultivate. We would therefore say to provincial students that, although the rivers of Damascus are undoubtedly pure and sweet, they are never likely to attain to the full virtues of the metropolitan Jordan. The provinces are able to build an admirable foundation, but London is still without rivals in the art of completing the superstructure.

